# HR23B pathology preferentially co-localizes with p62, pTDP-43 and poly-GA in *C9ORF72*-linked frontotemporal dementia and amyotrophic lateral sclerosis

**DOI:** 10.1186/s40478-019-0694-6

**Published:** 2019-03-13

**Authors:** Frederike W. Riemslagh, Hannes Lans, Harro Seelaar, Lies-Anne W. F. M. Severijnen, Shamiram Melhem, Wim Vermeulen, Eleonora Aronica, R. Jeroen Pasterkamp, John C. van Swieten, Rob Willemsen

**Affiliations:** 1 Department of Clinical Genetics, Erasmus University Medical Center Rotterdam, Rotterdam, The Netherlands; 2 Department of Molecular Genetics, Oncode Institute, Erasmus University Medical Center Rotterdam, Rotterdam, The Netherlands; 3 Department of Neurology, Erasmus University Medical Center Rotterdam, Rotterdam, The Netherlands; 4 Department of (Neuro)Pathology, Amsterdam Neuroscience, Amsterdam UMC, University of Amsterdam, Amsterdam, The Netherlands; 5 Department of Translational Neuroscience, University Medical Center Utrecht Brain Center, Utrecht University, Utrecht, The Netherlands

**Keywords:** *C9ORF72*, ALS, FTD, HR23B, ERAD, NGly1, DPRs, Poly-GA

## Abstract

Human homologue of yeast UV excision repair protein Rad23b (HR23B) inclusions are found in a number of neurodegenerative diseases, including frontotemporal dementia (FTD), Huntington’s disease (HD), spinocerebellar ataxia type 3 and 7 (SCA3/7), fragile X associated tremor/ataxia syndrome (FXTAS) and Parkinson’s disease (PD). Here, we describe HR23B pathology in *C9ORF72* linked FTD and amyotrophic lateral sclerosis (ALS) cases. HR23B presented in neuropils, intranuclear inclusions and cytoplasmic and perinuclear inclusions and was predominantly found in cortices (frontal, temporal and motor), spinal cord and hippocampal dentate gyrus. HR23B co-localized with poly-GA-, pTDP-43- and p62-positive inclusions in frontal cortex and in hippocampal dentate gyrus, the latter showing higher co-localization percentages. HR23B binding partners XPC, 20S and ataxin-3, which are involved in nucleotide excision repair (NER) and the ubiquitin-proteasome system (UPS), did not show an aberrant distribution. However, *C9ORF72* fibroblasts were more sensitive for UV-C damage than healthy control fibroblasts, even though all factors involved in NER localized normally to DNA damage and the efficiency of DNA repair was not reduced. HR23Bs other binding partner NGly1/PNGase, involved in ER-associated degradation (ERAD) of misfolded proteins, was not expressed in the majority of neurons in C9FTD/ALS brain sections compared to non-demented controls. Our results suggest a difference in HR23B aggregation and co-localization pattern with DPRs, pTDP-43 and p62 between different brain areas from C9FTD/ALS cases. We hypothesize that HR23B may play a role in *C9ORF72* pathogenesis, possibly by aberrant ERAD functioning.

**Electronic supplementary material:**

The online version of this article (10.1186/s40478-019-0694-6) contains supplementary material, which is available to authorized users.

## Introduction

The hexanucleotide (G_4_C_2_) repeat expansion in the chromosome 9 open reading frame 72 (*C9ORF72*) gene is the most common genetic cause of FTD and ALS [12, 33]. FTD is characterized by the degeneration of the frontal and temporal parts of the brain, leading to abnormalities in behavior, language and personality [49]. ALS affects motor neurons in the brain and spinal cord, leading to loss of motor function, muscle weakness, breathing problems and eventually paralysis [52]. Clinical, pathological and genetic factors connect FTD and ALS, and in families often patients present with symptoms of both disorders [4]. The discovery of the *C9ORF72* hexanucleotide repeat expansion confirmed the genetic overlap between FTD and ALS, collectively referred to as C9FTD/ALS. Pathologically, both diseases present with inclusions of autophagy protein p62/sequestosome 1 (p62) and inclusions of phosphorylated 43 kDa TAR DNA-binding protein (pTDP-43), of which the latter is predominantly found in areas that are known to display substantial neurodegeneration [24, 44].

The possible pathological mechanisms by which the *C9ORF72* repeat expansion can lead to FTD and ALS are: 1) hypermethylation of the repeat expansion and the CpG promoter region of the *C9ORF72* gene leading to haploinsufficiency [5, 43], 2) retention of repeat containing intron 1 in mRNAs causing RNA foci to appear in both nucleus and cytoplasm that sequester RNA-binding proteins [11, 27] or 3) the production of dipeptide repeats (DPR) by unconventional repeat-associated non-AUG (RAN) translation of the repeat [2, 18, 31]. These DPRs are produced from both sense and antisense transcripts resulting in 5 possibly toxic peptides (poly-GA, −GP, −GR, −PR and -PA) and are found as inclusions in post-mortem brain material of *C9ORF72* carriers [28, 38].

How these three mechanisms – alone or in combination – cause neurodegeneration is currently under investigation. Several studies have indicated that especially the DPRs are toxic in both cell culture and in vivo models, with the arginine-containing poly-GR and -PR DPRs being the most detrimental [3, 30, 47]. And poly-GR has been associated with neurodegeneration in human post-mortem brain sections [35, 36]. DPRs seem to cause various types of stress to the cell, including ER-stress, mitochondrial stress and nucleolar stress [3]. They can disturb the formation of membrane-less organelles, including RNA granules, nucleoli, spliceosomes and the nuclear pore complex (NPC) and facilitate the formation of stress granules [26]. Furthermore, nucleocytoplasmic transport and autophagy defects have been reported [40, 53, 56]. In addition to the list of disturbed pathways found in model systems of C9FTD/ALS, there have also been a substantial number of proteins found to interact, bind or aggregate with repeat-containing RNA or DPRs [21, 48]. Currently, the primary affected pathways involved in the pathogeneses of C9FTD/ALS are under debate [3, 17, 19]. Constituents of inclusions in human C9FTD/ALS brain material might provide a tool to identify key players in neurodegeneration.

Here, we tested a set of proteins implicated in aberrant pathways in *C9ORF72* disease models for their presence in pathology or abnormal localization in post-mortem human C9FTD/ALS brain sections. We selected Ran-GAP for its implication in nucleocytoplasmic transport defects [54], ADARB2 for its role in RNA binding and editing [13] and HR23B for its dual function in both DNA repair and the UPS [51]. FMRP and Pur-alpha were selected for their binding to *C9ORF72* mRNA, their localization in stress granules and their rescue effect in multiple *C9ORF72* models [34]. Surprisingly, we found only HR23B protein to be a constituent of inclusions observed in C9FTD/ALS cases. In this report, we describe HR23B distribution and its co-localization with known *C9ORF72* pathological hallmarks (DPRs, p62 and pTDP-43). Furthermore, we analyze HR23B function in DNA damage repair, the ubiquitin-proteasome system and ER-associated degradation. Disturbances of these pathways may contribute to the disease onset and/or progression of C9FTD/ALS.

## Materials and methods

Five *C9ORF72* FTD, two *GRN* FTD, two *MAPT* FTD, three sporadic ALS and three non-demented control human brain sections were provided by the Dutch Brain Bank. *C9ORF72* ALS brain material of two patients was collected post-mortem at the department of Neuropathology of Amsterdam UMC, University of Amsterdam, according to local legal and ethical regulations. Patients or relatives gave informed consent for autopsy and use of brain tissue for research purposes. Information about our patient cohort can be found in Table 1. Human fibroblasts lines were provided by the cell repository of the department of clinical genetics. All participants gave written informed consent for all obtained materials. The study was approved by the Medical and Ethical Review Committee of the Erasmus Medical Center. All procedures performed in studies involving human participants were in accordance with the ethical standards of the institutional and/or national research committee and with the 1964 Helsinki declaration and its later amendments or comparable ethical standards.

**Table 1:** Patient characteristics

Patient	Clinical diagnosis	Family history	Genetic diagnosis	Age of onset	Disease duration	Male/ Female	Brain weight
1	bvFTD	FTD	C9ORF72	51,8	8,7	Male	960 g
2	bvFTD	FTD	C9ORF72	55,8	9,1	Male	1184 g
3	bvFTD	FTD and ALS	C9ORF72	66,4	8,1	Female	1060 g
4	bvFTD	ALS and dementia	C9ORF72	63,2	6,8	Female	958 g
5	bvFTD	FTD and ALS	C9ORF72	55,2	9,5	Male	1075 g
6	FTD	N/A	Progranulin (Gln200X)	60,6	5,5	Female	894 g
7	FTD	FTD	Progranulin (Ser82ValfsX174)	47,4	4,3	Female	unknown
8	FTD	FTD	MAPT (G272 V)	42,6	8,4	Male	962 g
9	FTD	FTD	MAPT (P301L)	51,1	9,7	Male	887 g
10	ALS	N/A	unknown	70	1	Male	1428 g
11	ALS	N/A	unknown	65	2,2	Female	1125 g
12	ALS	N/A	unknown	75	1,1	Male	1255 g
13	ALS	N/A	C9ORF72	60	4,4	Female	1390 g
14	ALS	N/A	C9ORF72	66	3,5	Male	1275 g
15	ALS	N/A	C9ORF72	71	2,4	Female	1080 g
16	Non-demented	N/A	unknown	N/A	N/A	Female	1080 g
17	Non-demented	N/A	unknown	N/A	N/A	Male	1215 g
18	Non-demented	N/A	unknown	N/A	N/A	Female	1139 g

### Immunohistochemistry on human brain sections

Human brain sections (6 μm) were deparaffinized in xylene and rehydrated (100%–96%-90%–80%-70%–50% EtOH serie). Antigen retrieval was done in 0.01 M sodium citrate, pH 6.0 using pressure cooker treatment. Endogenous peroxidase activity was blocked with 0.6% H_2_O_2_ and 1,25% sodium azide in 0.1 M PBS. Immunostaining was performed overnight at 4 °C in PSB block buffer (0.1 M PBS/0.5%protifar/0.15%glycine). Antibodies used in this study are listed in Additional file 1: Table S3, including concentration and brand/catalogue number. Antigen-antibody complexes were visualized by incubation with DAB substrate (DAKO) after incubation with Brightvision poly-HRP-linker (Immunologic) or anti-mouse/rabbit HRP (DAKO). Slides were counterstained with Mayer’s haematoxylin and mounted with Entellan (Merck Millipore International). The slides were then left to dry in the fume hood for an hour and thereafter put in an 37 °C incubater overnight. Pictures were taken by using an Olympus BX40 microscope (Olympus).

### Immunofluorescence staining on human brain sections

Human brain sections were treated as described above. After incubation with the primary antibody, sections were washed with PBS block buffer (1xPBS/0.5%protifar/0.15%glycine) and incubated with secondary anti-mouse/rabbit Cy2/3 linked antibodies (Jackson). To remove background staining, a 10 min incubation with Sudan Black (Sigma, 0.1 g in 100 ml 70% ethanol, filtered) was done. To visualize nuclei, slides were incubated for 10 min with Hoechst 33342(Invitrogen). Slides were mounted with ProLongGold (Invitrogen) and kept at 4 °C until imaging at a Zeiss LSM700 Confocal microscope.

### Assessment of neurodegeneration and protein pathology

For the neuropathological assessment we used brain sections from 5 *C9ORF72* FTD cases (see Table 1). Five different brain regions (frontal cortex, temporal cortex, motor cortex, hippocampus and cerebellum) per patient were semi-quantified on neurodegeneration and pathological score of p62, pTDP-43 and HR23B (Additional file 2: Table S1). Counting was not performed in a blinded fashion. Neurodegeneration was assessed on haematoxylin and eosin (HE) sections and graded as absent (0), mild (1), moderate (2) or severe (3) based on the presence of neuronal loss. The neurodegenerative score from the pathological report was also taken into account. Pathological scores were rated as (0) if completely absent, rare (1) if only a few could be found one brain section, occasional (2) if they not present in every microscopic field, moderate (3) if at least a few examples were present in most microscopic fields, and numerous (4) when many were present in every microscopic field [28]. We reported the overall number of immunoreactive inclusions (total score) as well as the number of neuronal cytoplasmic inclusions (NCI), neuronal intranuclear inclusions (NII) and dystrophic neurites (DN) (Additional file 2: Table S1). Total scores were assessed independent from NCI, NII and DN scores and represent an impression of the global load of pathology and the number of inclusions using the same grading system as described above. Quantification of co-localization of HR23B with DPRs, p62 and pTDP-43 was not performed in a blinded fashion.

### Colony forming assays

Human fibroblast lines from 4 *C9ORF72* carriers (13E634, 13E659, 17E0225, 17E0278) and 4 controls (81E253, 86E1375, 06E0717 and 99E0774) were obtained from the cell repository of the department of clinical genetics and the XP25RO fibroblast line was provided by the department of molecular genetics. Fibroblasts were cultured in DMEM medium (Gibco) with 10% fetal calf serum, 1% penicillin/streptavidin and 1% non-essential amino acids. To determine UV-sensitivity, human fibroblasts were seeded in triplicate in 10 cm plates (Greiner Bio-one) in a density of 2000 cells/plate. After 24 h, cells were treated with increasing doses of UV-C (254 nm UV-C lamp, Philips). After 5–7 days, colonies were fixed with 0.1% *w*/*v* Coomassie Blue (Bio-Rad) in a 50% Methanol, 10% Acetic Acid solution. Colonies were counted with the integrated colony counter GelCount (Oxford Optronix). Counting was performed automatically with the same settings for each fibroblast cell line, but not in a blinded fashion.

### Immunofluorescence on human fibroblasts

To determine DNA damage recruitment of NER proteins, human fibroblasts were seeded on coverslips and after 1 week in culture irradiated with 60 J/m2 (254 nm UVC lamp, Philips) through an 8 μm microporous filter (Millipore) to induce sub-nuclear local DNA damage. Cells were fixed after 30 min with 2% paraformaldehyde and permeabilized with 0.1% Triton X-100 for 20 min. Next, cells were incubated with fresh 0.07 M NaOH in PBS for 5 min to denature DNA and enable CPD staining. Cells were then washed with PBS containing 0.15% glycine and 0.5% BSA and incubated with primary antibodies (see Additional file 1: Table S3) overnight at 4 °C. The next day, cells were washed with 0.1% Triton X-100 and incubated with Alexa Fluor conjugated secondary antibodies (488, 555 and 633; Invitrogen) for 1 h at RT. Coverslips were mounted using ProLongGold with DAPI (Invitrogen) and imaged using a Zeiss LSM700 microscope.

### Unscheduled DNA synthesis (UDS)

To measure NER capacity, human fibroblasts were grown on coverslips for 1 week in culture and irradiated with 16 J/m^2^ UV-C. After irradiation, cells were incubated for 1 h in UDS medium (F10, 1% PS, 10% dialyzed serum) with 2% HEPES and 1% 5-ethynyl-2′-deoxyuridine (EdU, Invitrogen). After 1 h, medium was changed to normal DMEM medium (10% FCS, 1% NEAA, 1% PS) for 10 min. Next, cells were fixed in 4% paraformaldehyde and permeabilized with 0.1% Triton X-100. Blocking was done using 1.5% BSA in PBS for 30 min. EdU incorporation was visualized by incubating cells for 1 h at room temperature with Click-it reaction cocktail containing Atto 594 Azide (60 μM, Atto Tec.), Tris-HCl (50 mM, pH 7.6), CuSO_4_*5H_2_O (4 mM, Sigma) and ascorbic acid (10 mM, Sigma). After the Click-it reaction, cells were washed with 0.1% Triton X-100, incubated with Hoechst 33342 (Invitrogen) for 10 min and mounted with ProLongGold (Invitrogen). Images were acquired using a Zeiss LSM700 microscope. UDS levels were quantified by measuring the total nuclear fluorescence intensities (in at least 50 cells per experiment) with FIJI image analysis software. Intensity levels were averaged and normalized to the fluorescence levels in unirradiated cells. Quantification of intensity levels was done automatically with FIJI but was not performed in a blinded fashion.

## Results

### Characterizing Ran-GAP, ADARB2, HR23B, FMRP and Pur-alpha in a cohort of C9FTD patients and non-demented controls

We started with assessing the localization of ras-related nuclear protein GTPase activating protein (Ran-GAP) in C9FTD patient brain sections. We studied five FTLD-TDP cases with the *C9ORF72* repeat expansion and three non-demented cases (for information about our cohort, see Table 1). We predominantly found a diffuse nuclear staining or nuclear envelop staining of Ran-GAP. Most nuclei were round-shaped but occasionally we observed misfolded nuclei or the invagination of the nuclear membrane (Additional file 3: Figure S1). The number of oddly-shaped nuclei did not clearly differ between C9FTD cases and non-demented controls. Next, we investigated Adenosine Deaminase, RNA Specific B2 (ADARB2) localization. ADARB2 immunostaining showed both nuclear and cytoplasmic localization in both C9FTD and non-demented controls. We could detect some intranuclear inclusions in the hippocampus dentate gyrus of C9FTD patients (Additional file 4: Figure S2A). However, IF double staining of ADARB2 with p62 showed similar results between hippocampal nuclei of C9FTD patients and non-demented cases (Additional file 4: Figure S2B).

**Fig. 1:**
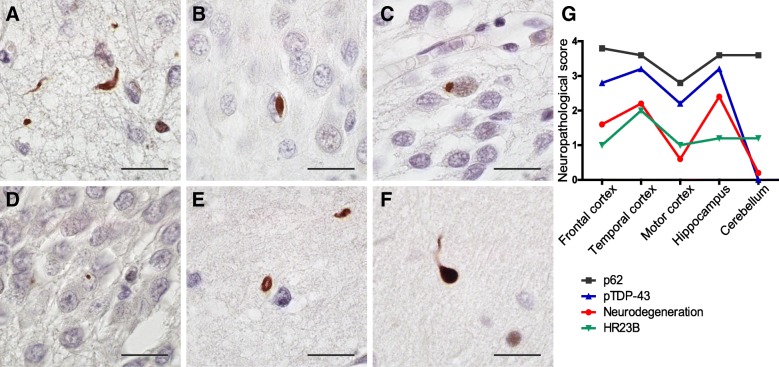
Type and spreading of HR23B pathology found in C9FTD cases. Different types of HR23B pathology in C9FTD cases: **a**) neuropils and puncta in frontal cortex layer 2. **b**) intranuclear (cat eye) inclusion in hippocampus dentate gyrus. **c**) perinuclear inclusion in hippocampus dentate gyrus. **d**) round intranuclear inclusion in hippocampus dentate gyrus. **e**) round or oval inclusion with a hole in frontal cortex **f**) dystrophic neuron in cerebellum molecular layer. **g**) Spreading of HR23B compared to known p62 and pTDP-43 pathology. Depicted are semi-quantitive measures of neurodegeneration and pathological score in C9FTD. Neuronal loss score was based on hematoxylin and eosin (HE) staining and pathological report and scored as absent (0), mild (1), moderate (2) or severe (3). Pathological scores were based on the degree of pathology as absent (0), rare (1), occasional (2), moderate (3), or numerous (4). See also Additional file 9: Table S2 for details of pathological quantifications. All scale bars are 20μm

Next, we stained for HR23B and found different types of inclusions in cortical areas, hippocampus and cerebellum of C9FTD cases (Fig. 1 and Additional file 5: Figure S5). Most cytoplasmic inclusions showed a round and perinuclear appearance (Fig. 1a and c), while some inclusions had a negative central core surrounded by a positive halo labeling (Fig. 1e). Neuropils were mostly found in layer 2 of cortical areas (Fig. 1a). Intranuclear inclusions were also observed, including cat-eye inclusions (Fig. 1b). Overall, layer 2/3 and layer 5/6 of frontal and temporal cortices showed the highest HR23B pathological burden in C9FTD cases, followed by motor cortex, hippocampus and cerebellum (Fig. 1g and Additional file 2: Table S1). Hippocampus dentate gyrus (DG) harbored some perinuclear inclusions and hippocampus cornu ammonis (CA) showed some cells with strong nuclear staining (Additional file 5: Figure S5). Cerebellum showed low HR23B staining with some nuclear and perinuclear inclusions in the granular layer and nearly absent in the molecular layer (Additional file 5: Figure S5). Non-demented controls showed normal nuclear localization of HR23B (Additional file 5: Figure S5).

Furthermore, we assessed fragile X mental retardation protein (FMRP) localization in C9FTD cases. Punctuated FMRP staining in the cytoplasm indicative of the formation of stress granules was absent in our C9FTD cases (Additional file 6: Figure S3). FMRP was evenly distributed in the cytoplasm in both C9FTD cases and controls. Very occasionally, we could detect inclusions in the hippocampus dentate gyrus in both C9FTD cases and controls (Additional file 6: Figure S3). Finally, we set out to test Pur-alpha staining, which showed to be located in stress granules in both C9FTD cases and controls (Additional file 7: Figure S4). We did not find Pur-alpha intranuclear inclusions in the cerebellum or other brain areas of C9FTD cases nor controls (Additional file 7: Figure S4).

### HR23B pathology is also present in C9ALS and *GRN* FTD post-mortem brain tissue

**Fig. 2:**
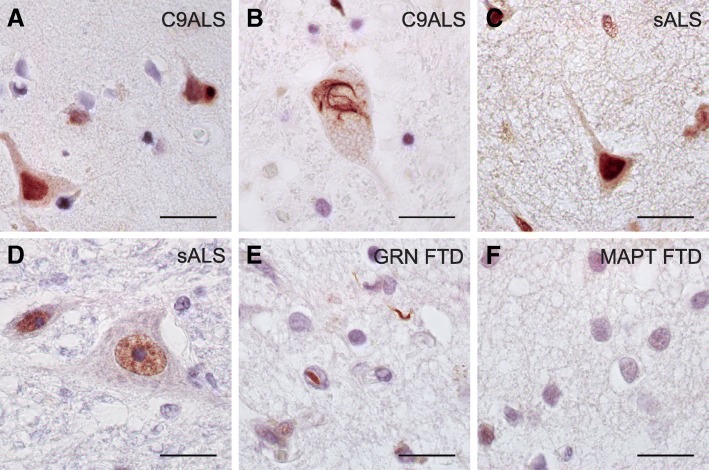
HR23B pathology is also present in C9ALS and *GRN* FTD cases. **a**) HR23B staining in C9ALS motor cortex shows strong staining in the nucleus and a cytoplasmic inclusion. **b**) C9ALS spinal cord section with cytoplasmic HR23B pathology **c**) Sporadic ALS with very strong nuclear HR23B staining in motor cortex **d**) and in spinal cord. **e**) *GRN* FTD case with an intranuclear inclusion and neurites positive for HR23B in frontal cortex. **f**) HR23B pathology is absent in frontal cortex of *MAPT* FTD. All scale bars are 20 μm

In order to validate the observed HR23B pathology, we used a second independent HR23B antibody (for details see methods) that revealed similar results (Additional file 8: Figure S6). Furthermore, we expanded our cohort with three ALS cases with the *C9ORF72* repeat expansion, three sporadic ALS cases with unknown genetic cause, two FTD with *GRN* mutation and two FTD-*MAPT* cases. In non-demented controls, we observed immunoreactivity for HR23B in nuclei (Additional file 5: Figure S5 and Additional file 8: Figure S6). C9ALS cases showed very strong HR23B staining in nuclei and some cytoplasmic inclusions in motor cortex (Fig. 2a) and spinal cord sections (Fig. 2b). Sporadic ALS cases also showed very strong nuclear HR23B staining but no pathology in motor cortex (Fig. 2c) nor spinal cord (Fig. 2d). *GRN* FTD cases showed the same extend of HR23B pathology as C9FTD in frontal cortex, consisting of some intranuclear inclusions, cytoplasmic inclusions and neuropils (Fig. 2e). HR23B pathology was absent in *MAPT* FTD cases (Fig. 2f).

### HR23B co-localizes with poly-GA, pTDP-43 and p62 in C9FTD cases

**Fig. 3:**
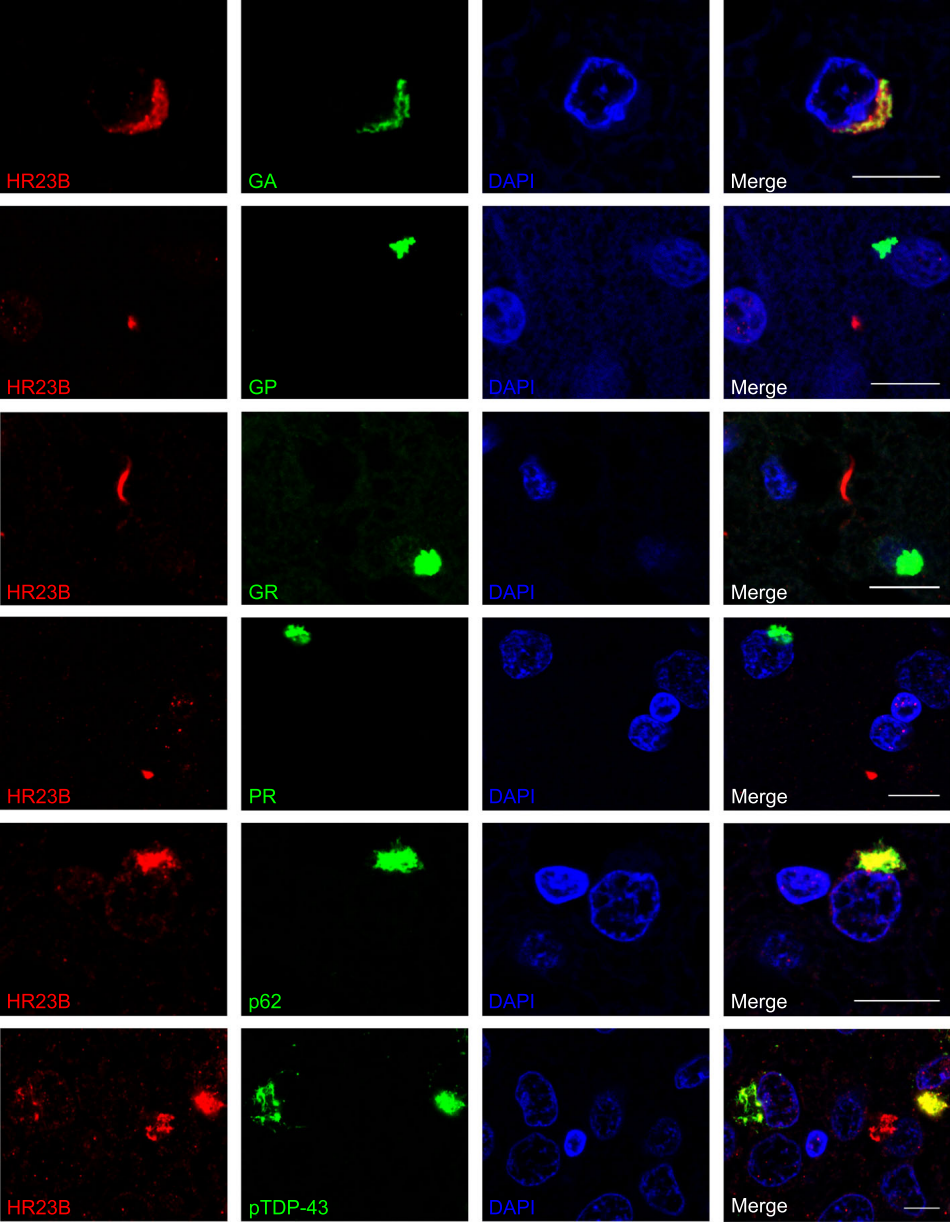
HR23B co-localizes with p62, TDP-43 and poly-GA in C9FTD cases. Immunofluorescent staining for HR23B (shown in red) in combination with DPRs (poly-GA, −GP, −GR and -PR) or p62 or pTDP-43 (shown in green). Poly-PA was not evaluated because too few inclusions were found. All pictures are from frontal cortex of C9FTD cases. All scale bars are 10 μm

**Fig. 4:**
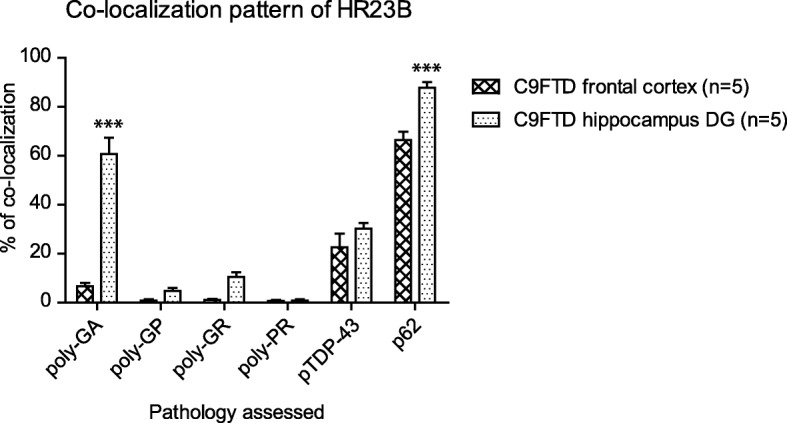
HR23B co-localization percentages with poly-GA and p62 differ between frontal cortex and hippocampus DG. Semi-quantification of co-localization of HR23B with pathological hallmarks such as DPRs, p62 and pTDP-43 based on raw data in Additional file 9: Table S2. Two-way ANOVA is significant (*p* < 0.0001) for pathology, brain area and interaction. Bonferroni test indicates that only poly-GA and p62 are significantly different between frontal cortex and hippocampus DG (both *p* < 0.001)

To evaluate HR23B’s aggregation process in C9FTD, we performed double labeling with known pathological hallmarks, including DPRs, pTDP-43 and p62 in five C9FTD frontal cortices (Fig. 3). HR23B was mostly found to be co-localized with p62 (Fig. 3), as 66% of all HR23B inclusions were also positive for p62 (Additional file 9: Table S2). Next is pTDP-43 with 22.6% of HR23B inclusions being positive for pTDP-43 (Fig. 3 and Additional file 9: Table S2). From all DPRs, HR23B showed partial co-localization with poly-GA (Fig. 3), for 6.6% (Additional file 9: Table S2). The other DPRs only co-stained with 0–3% of all HR23B inclusions. We did not evaluate poly-PA because we found too few inclusions. Interestingly, hippocampus dentate gyrus showed a much higher co-localization between DPRs and HR23B than frontal cortex (Fig. 4 and Additional file 9: Table S2). We found 60.6% of HR23B inclusions being positive for poly-GA in the dentate gyrus, which is nearly a 10-fold increase compared to the frontal cortex (Fig. 4 and Additional file 9: Table S2). Poly-GP increased from 0.8% in frontal cortex to 4.7% in hippocampus and poly-GR also showed a 10-fold increase from 1% in frontal cortex to 10.5% in hippocampus (Fig. 4 and Additional file 9: Table S2). Only poly-PR stayed fairly undetectable with a slight increase from 0.65% in frontal cortex to almost 1% in hippocampus. HR23B co-localization with p62 in dentate gyrus was also higher in hippocampus than in frontal cortex (87.4% vs 66%) (Fig. 4 and Additional file 9: Table S2). We performed a 2-way ANOVA to compare co-localization percentages of HR23B with pathological hallmarks between different brain areas (*p* < 0.0001). Post Bonferroni test indicated that percentages of poly-GA and p62 were significantly different between hippocampus DG and frontal cortex (Fig. 4 and Additional file 9: Table S2). For the other DPRs and pTDP-43, differences were not significant (Fig. 4 and Additional file 9: Table S2). These data suggest a difference in aggregation formation and co-localization patterns between different brain areas or cell types.

### Nucleotide excision repair is not affected in C9ORF72 patient fibroblasts

**Fig. 5:**
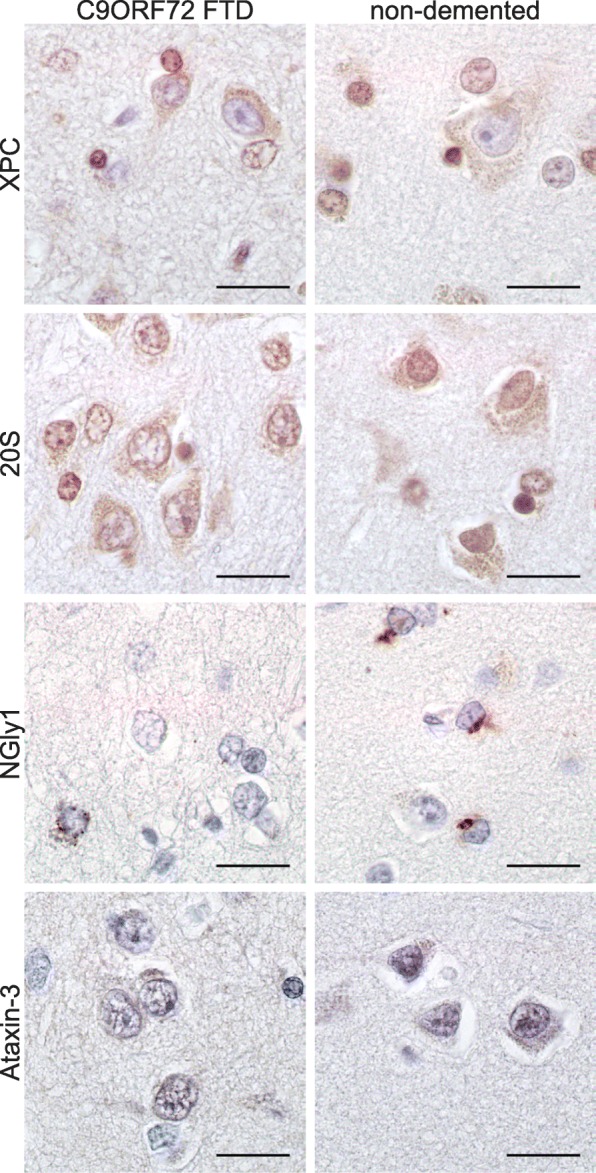
HR23B does not sequester its bindings partners into inclusions. XPC, 20S and ataxin-3 stainings do not reveal any differences between C9FTD patients and non-demented controls. For NGly1, we observed less nuclei with perinuclear staining in *C9ORF72* FTD brains than in non-demented controls. All pictures are from frontal cortex. All scale bars are 20 μm

To assess any changes in the normal cellular function of HR23B, we first focused on its role in DNA damage repair. HR23B interacts with and stabilizes Xeroderma pigmentosum, complementation group C (XPC) protein [32], which is involved in the recognition of bulky DNA adducts in global genome nucleotide excision repair (GG-NER). Staining with XPC antibody did not reveal any gross differences between C9FTD and non-demented control post-mortem brain sections (Fig. 5). To assess nucleotide excision repair capacity in living cells, we performed UV-sensitivity assays with four *C9ORF72* patient fibroblast lines and compared these to four healthy control fibroblast lines. Intriguingly, *C9ORF72* fibroblasts were more sensitive to UV-C damage than healthy control fibroblasts (Additional file 10: Figure S7A), but not as sensitive as a fibroblast line that is fully deficient in NER (XP25RO, homozygous for 619C > T causing an ARG207X change in exon 5 of the XPA gene). The GG-NER pathway is initiated by recognition of DNA damage by the HR23B/XPC/CETN2 complex, which is then followed by multiple downstream steps to verify and excise the damage [29]. We therefore assessed whether factors involved in each of the steps of the NER pathway were recruited normally to DNA damage in *C9ORF72* fibroblasts. To do so, we evoked local DNA damage by UV-C irradiation through a microporous filter in our fibroblast lines and performed immunofluorescence to visualize DNA damage (by cyclobutane pyrimidine dimers (CPD) antibody) and DNA damage recruitment of NER factors XPC, XPB, XPA, XPF and XPG. All tested NER proteins clearly co-localized with DNA damage in *C9ORF72* patient fibroblasts, indicating that recognition and processing of CPDs is still functional in these cells (Additional file 10: Figure S7B). To finally verify that NER is fully operational, we measured incorporation of the thymidine analogue 5-ethynyl-2-deoxiuridine (EdU) after UV-C irradiation to quantify the efficiency of DNA repair in our fibroblast lines. The NER-deficient XP25RO cell line, which we used as negative control, did not show any EdU incorporation. In contrast, we still observed efficient EdU incorporation in our four C9ORF72 patients cell lines to similar levels as in four healthy control lines (Additional file 10: Figure S7C), indicating that NER is not deficient in *C9ORF72* patient fibroblasts.

### NGly1, ERAD factor and HR23B binding partner, is less abundant in C9FTD brain sections

Besides its role in DNA damage repair, HR23B is also known for its function in the ubiquitin-proteasome system. Various HR23B binding partners have been identified which are involved in the unfolded protein response (UPR), transcriptional regulation, cell cycle control and ER-associated degradation (ERAD) [51]. To assess if HR23B aggregation also evokes changes in the localization of the proteasome, we stained our sections for proteasome subunit 20S. However, we did not observe any obvious changes in 20S normal localization (Fig. 5). Another binding partner of HR23B is ataxin-3, a deubiquitinase enzyme in which a poly-glutamine expansion is linked to SCA3 [46]. HR23B-positive inclusions have been found in post-mortem brain material of SCA3 patients [6] however we could not detect ataxin-3 pathology in C9FTD cases (Fig. 5). Besides the proteasome, we wondered if we could find any changes in ERAD. HR23B did not sequester NGly1, one of its known binding partners involved in ERAD, into protein inclusions. Strikingly, however, we did observe clearance of NGly1 staining in C9FTD frontal cortex (Fig. 5). Although some neurons showed the same strong peri-nuclear staining as almost all non-demented control cells, in the majority of patient neurons no NGly1 staining was observed, indicating reduced expression of NGly1 in C9FTD brains. This may suggest a partial loss of function of ERAD in a subset of neurons in the brain of C9FTD patients.

## Discussion

In the present study, we characterize HR23B pathology distribution and its co-localization pattern with pathological hallmarks. To our knowledge, we are the first to show that HR23B co-localizes with pTDP-43 pathology in brain tissue of C9FTD patients. We could also demonstrate HR23B pathology in C9ALS and sporadic ALS. HR23B pathology has been described before in HD, SCA3, SCA7, FXTAS and PD [6]. HR23B pathology was also present in both *C9ORF72* and *GRN* FTD cases, but not in *MAPT* FTD cases, in contrast to a previous report of HR23B pathology described for FTDP-17 (FTD with parkinsonism with Pick bodies consisting of tau protein) [6]. Also Alzheimer’s disease (AD) brain material did not show HR23B pathology [6]. Why AD and some FTLD-tau patients are an exception and do not present with HR23B pathology is unclear and requires further investigation. It should be noted that our study cohort was rather small (see Table 1), which might explain why we could not confirm HR23B pathology in *MAPT* FTD cases. Next to HR23B, HR23A inclusions have been reported in FXTAS and in C9FTD post-mortem brain tissue [6, 55]. In general, HR23 pathology seems to be widespread among neurodegenerative diseases, which underscores its relevance in a common disease pathogenesis of these disorders.

In our semi-quantitative co-localization studies, we found HR23B to be co-localized predominantly with p62, followed by pTDP-43 and poly-GA. HR23B inclusions were nearly always negative for the other DPRs. Overexpression of poly-GA in mouse models is enough to sequester HR23B [37, 55] and in a co-immunoprecipitation experiment of lysates from cells expressing GFP-tagged poly-GP, −GR and -GA, only poly-GA was found to bind HR23B [55]. This could also be a stochastic event, given the fact that poly-GA inclusions are the most abundant DPR in C9FTD/ALS brains [28]. It could also be caused by the aggregation-prone nature of the poly-GA peptide itself [9], causing HR23B to bind easier or faster to poly-GA peptides than other DPRs. Binding of HR23B to DPRs also differs between brain areas; in the frontal cortex, only 6.6% of HR23B inclusions were poly-GA positive, while this increased to 60.6% in the hippocampus dentate gyrus. The same is true for poly-GP and -GR; co-localization with HR23B increased 5- to 10-fold between frontal cortex and hippocampus, although it remained low (1–10%). Subtypes of neurons may differ in their vulnerability for DPRs or there might be differences in the expression level and availability of HR23B and/or its binding partners between frontal cortex and hippocampus dentate gyrus. Levels of HR23B can influence aggregation of poly-GA and toxicity of mutant TDP-43 and mutant SOD1 [23, 55]. Overexpression of HR23B protected for the formation of poly-GA inclusions in mouse primary neuronal cultures [55]. In contrast, loss of HR23B seems to protect against motor neuron disease by enhancing mutant protein clearance [23]. Levels of HR23B seem to have opposite effects on aggregation, clearance and solubility of several proteins, which could result in differences in aggregation and co-localization patterns observed between different brain areas.

HR23B can directly bind the proteasome and ataxin-3, a deubiquitinase enzyme that binds ubiquitinated proteins and can also bind to the proteasome [14, 46]. Even though HR23B and proteasome subunits are sequestered into intranuclear inclusions of ataxin-3 in SCA3 patients brain tissue [6, 39], ataxin-3 and 20S do not show an aberrant localization in C9FTD patients post-mortem brain tissue. HR23B can also bind PNGase, a deglycosylation hydrolase involved in ERAD of misfolded glycoproteins. The affinity of PNGase for the proteasome is HR23B-dependent, which makes HR23B essential for the shuttling of misfolded proteins to the proteasome [25]. If HR23B is sequestrated into inclusions and becomes unavailable for PNGase, this might cause loss of initiation of ERAD. This is in line with our observation that a substantial number of neurons of C9FTD/ALS patients show less abundant NGly1 staining. Many FTD-causing mutations are associated with protein degradation pathways [20]. In addition, mutations in *NGLY1*, the gene encoding PNGase, are linked to motor impairment, intellectual disability, and neuropathy in humans [8].

HR23B is well known for its role in global genome nucleotide excision repair (GG-NER) and genetic polymorphisms in *RAD23B* are modifiers of laryngeal cancer risk in human [1]. The DNA damage response can be induced by the *C9ORF72* repeat expansion [15] and elevated levels of R-loops (DNA-RNA hybrids), double strand breaks and ATM-mediated DNA repair signaling defects have been described before in rat neurons, human cells and C9ALS spinal cord tissue [15, 45]. Furthermore, ALS and *C9ORF72* repeat carriers have an increased risk for melanoma [16, 42], suggesting they may have an reduced response to DNA damage. XPC, the binding partner of HR23B in NER, was found in inclusions in a poly-GA mouse model of C9FTD/ALS [55]. Nonetheless, we could not find XPC pathology in our human brain sections nor deficits in the NER pathway in *C9ORF72* patient fibroblasts, even though *C9ORF72* patient fibroblasts seem to be more sensitive for UV-C damage than healthy control fibroblasts. Why we do not find a clear impairment of NER in our study is unknown. Species-specific factors, overexpression of poly-GA in the mouse model or difference between fibroblasts and neurons might explain a part of the absence of an effect. In addition, it could be possible that HR23A takes over the DNA repair function of HR23B when the latter is sequestered or dysfunctional. This has been demonstrated in *mHr23b* knockout (KO) mice that show no impairment in NER [51]. Still, *mHr23b* KO mice display impaired embryonic development, retarded growth and facial dysmorphologies that are not observed in mouse models deficient in other NER genes [51], which suggests a second function of HR23B. Although HR23A and HR23B have similar functions in DNA repair, they form distinct interactions with various cellular factors, including proteasomes, multi-ubiquitinated proteins and stress-related factors [10].

Here, we set out to validate the aggregation of several proteins that have been described to mis-localize or bind RNA foci in C9FTD/ALS. Strikingly, we could not reproduce earlier published pathology for Ran-GAP, ADARB2, Pur-alpha and FMRP. The differences observed between our study and previous publications can be explained by multiple factors. First of all, we used post-mortem brain material that only presents the end-stage of the disease, so changes in localization of proteins in early stages of the disease can be missed. Also, the number of cells presenting with stress granules varies a lot between subjects and might be attributed to autolytic processes during human brain preservation, which can make it hard to detect subtle differences. Secondly, this study focused on FTD rather than ALS. This could especially be important for ADARB2, as one of the targets of ADAR proteins is the Q/R site of the GluR2 AMPA receptor [22]. Changes in ADARB2 localization could therefore mostly affect ALS cases and may be missed in our FTD cohort. Thirdly, pathology observed in cell culture and in vivo models could be due to overexpression of *C9ORF72* RNA or DPRs in these models. Changes in patient neurons with endogenous expression can be more subtle but still act disturbing over time. Most model systems used so far do not include haploinsufficiency, which can be a modifying factor for cellular toxicity as well [41]. Finally, effects might be missed due to our small cohort. For example, differences in oddly-shaped nuclei in the Ran-GAP staining might only become evident when quantifying large number of cells. However, Saberi et al. also were unable to confirm Ran-GAP pathology [35], which strengthens our findings and illustrates the need for validation studies in biomedical research. Even though we did not observe any pathology of Ran-GAP, ADARB2, FMRP and Pur-alpha, their levels could still have a modifying effect on disease progression of FTD, as has been shown in iPSC-derived neurons and Drosophila models [7, 13, 50, 54].

## Conclusion

In this study, we confirm HR23B aggregation and its implication in C9FTD/ALS. HR23B has an important function in both the DNA damage response and the degradation of proteins via the UPS, UPR and ERAD. Our results in human postmortem brain tissue suggests that especially the degradation of proteins via ERAD may be involved in the pathogenesis of ALS and FTD. The exact role and timing of HR23B in disease onset and progression needs further investigation, including its interaction with and possible degradation of proteins implicated in neurodegenerative disorders.

## Additional files

Additional file 1: **Table S3.** Antibody information. (DOCX 16 kb)

Additional file 2: **Table S1.** Neuropathological scores of *C9ORF72* FTD patients. Neuronal loss score was based on hematoxylin and eosin (HE) staining and pathological report and scored as absent (0), mild (1), moderate (2) or severe (3). Pathological scores were based on the degree of pathology as absent (0), rare (1), occasional (2), moderate (3), or numerous (4). Brain areas: F = frontal cortex, T = temporal cortex, M = motor cortex, H = hippocampus dentate gyrus, C = cerebellum. NCI = neuronal cytoplasmic inclusion, NII = neuronal intranuclear inclusion, DNs = dystrophic neurites. (DOCX 23 kb)

Additional file 3: **Figure S1.** Ran-GAP staining in C9FTD cases and non-demented controls. Ran-GAP is predominantly localized to the nucleus and nuclear membrane. Unevenly shaped nuclear membranes occur in both *C9ORF72* FTD cases (*n* = 5) and non-demented controls (*n* = 3). All scale bars are 20 μm. (PDF 2202 kb)

Additional file 4: **Figure S2.** ADARB2 staining in C9FTD cases and non-demented controls. A) Staining of ADARB2 in *C9ORF72* FTD cases (n = 5) and non-demented control (n = 3) post-mortem brain sections shows some intranuclear inclusions in hippocampus CA and DG. All scale bars are 20 μm B) Immunofluorescence staining of ADARB2 (red) and p62 (green) in hippocampal dentate gyrus reveals ADARB2 punctuated staining and some intranuclear inclusions in both *C9ORF72* FTD cases (n = 5) and non-demented controls (n = 3). Scale bars in fluorescent pictures are 10 μm. (PDF 2721 kb)

Additional file 5: **Figure S5.** HR23B pathology in different brain areas of C9FTD cases. Staining of HR23B in several brain areas of C9FTD cases and non-demented controls. Pathology burden was highest in cortices (frontal, temporal and motor) and was mostly cytoplasmic (inclusions and neuropils) and intranuclear (cateye). Hippocampus dentate gyrus (DG) harbors perinuclear inclusions, and hippocampus cornu ammonis (CA) had some cells with strong nuclear staining. Pathology was low in cerebellum granular layer with only some nuclear and perinuclear inclusions and almost absent in cerebellum molecular layer. Our C9FTD cases did not show HR23B pathology in spinal cord neurons. All scale bars are 20 μm (PDF 2260 kb)

Additional file 6: **Figure S3.** FMRP staining in C9FTD cases and non-demented controls. FMRP staining does not reveal any differences between *C9ORF72* FTD cases (n = 5) and non-demented control (n = 3) post-mortem brain sections. Occasional inclusions are found in the hippocampus dentate gyrus in both C9FTD cases and controls. All scale bars are 20 μm. (PDF 1883 kb)

Additional file 7 **Figure S4** Pur-alpha staining in C9FTD cases and non-demented controls. Pur-alpha staining reveals abundant stress granules in both *C9ORF72* FTD cases (n = 5) and non-demented controls (n = 3) post-mortem brain sections. All scale bars are 20 μm. (PDF 1826 kb)

Additional file 8 **Figure S6** Validation of HR23B pathology by a second independent antibody. HR23B staining using Abcam antibody in *C9ORF72* FTD cases (n = 5) and non-demented control (n = 3) post-mortem brain sections. Staining pattern is consistent with HR23B GeneTex antibody (see Figs. 1 and 2). All scale bars are 20 μm (PDF 1543 kb)

Additional file 9: **Table S2.** Co-localization of HR23B with DPRs/pTDP-43/p62 differs between brain areas. The percentage is the amount of HR23B inclusions also positive for other pathological hallmarks (not the other way around). For example 11/153 for poly-GA in C9FTD patient 1 means that out of 153 HR23B inclusions, 11 were also positive forpoly- GA, which is 7%. F = frontal cortex. H = hippocampus dentate gyrus. *1 = All co-localizations are fibrils of poly-GP and HR23B in frontal cortex. Perinuclear inclusions of poly-GP did not stain positive for HR23B. *2 = In total 2 poly-PA inclusions have been found in frontal cortex of 5 C9FTD patients, too less to quantify. We therefore state N/A = non-applicable in this table. *3 = Non-demented cases had some p62 and some HR23B inclusions per person per section, which sometimes overlapped. No pTDP-43 inclusions were found so no co-localization of pTDP-43 with HR23B in non-demented cases. (DOCX 19 kb)

Additional file 10: **Figure S7.** Nucleotide excision repair is not affected in *C9ORF72* human fibroblasts. A) Dose-response curve for 4 healthy control human fibroblast lines (81E253, 86E1375, 06E0717 and 99E0774) and 4 *C9ORF72* human fibroblast lines (13E634, 13E659, 17E0225, 17E0278) and the NER deficient XP25RO human fibroblast line treated with increasing dose of UV-C light (0–12 J/m2). B) Immunofluorescence staining showing the recruitment of NER factors XPC, XPB, XPA, XPF and XPG to local DNA damage (visualized by CPD antibody), induced by 60 J/m2 UV-C irradiation through a microporous filter. Fibroblast lines used for pictures: 13E634, 13E659 and 81E253 C) Human fibroblasts lines were treated with 16 J/m2 UV-C light and incubated with EdU for 1 h to measure unscheduled DNA synthesis (UDS) as measure of DNA repair. The NER-deficient XPC25RO cell line is shown as negative control. Ctrl 1–4 are lines 81E253, 86E1375, 06E0717 and 99E0774 in this order. C9 1–4 are lines 13E634, 13E659, 17E0225, 17E0278 in this order. (PDF 934 kb)

## Abbreviations

AD: Alzheimer’s disease
ADARB2: Adenosine Deaminase, RNA Specific B2
ALS: Amyotrophic lateral sclerosis
C9ORF72: Chromosome 9 open reading frame 72
CPD: Cyclobutane pyrimidine dimers
DN: Dystrophic neurites
DPRs: Dipeptide repeat proteins
EdU: 5-ethynyl-2-deoxiuridine
ERAD: Endoplasmic reticulum associated degradation
FMRP: Fragile X mental retardation protein
FTD: Frontotemporal dementia
FXTAS: Fragile X associated tremor/ataxia syndrome
GG-NER: Global genome nucleotide excision repair
HD: Huntington’s Disease
HE: Haematoxylin and eosin
HR23B: Human homologue of yeast UV excision repair protein Rad23b
IF: Immunofluorescence
IHC: Immunohistochemistry
NCI: Neuronal cytoplasmic inclusions
NCT: Nucleocytoplasmic transport
NER: Nucleotide excision repair
NII: Neuronal intranuclear inclusion
NPC: Nuclear pore complex
p62: Sequestosome 1
PD: Parkinson’s disease
poly-GA: Poly-glycine-alanine
poly-GP: Poly-glycine-proline
poly-GR: Poly-glycine-arginine
poly-PA: Poly-proline-alanine
poly-PR: Poly-proline-arginine
pTDP-43: Phosphorylated 43 kDa TAR DNA-binding protein
RAN: Repeat-associated-non-AUG
Ran-GAP: ras-related nuclear protein GTPase activating protein
SCA3/7: Spinocerebellar ataxia type 3 and 7
UDS: Unscheduled DNA Synthesis
UPR: Unfolded-protein response
UPS: Ubiquitin-proteasome system
XPC: Xeroderma pigmentosum complementation group C
