# Supplementary material for: HR23B pathology preferentially co-localizes with p62, pTDP-43 and poly-GA in C9ORF72-linked frontotemporal dementia and amyotrophic lateral sclerosis
Source: Acta Neuropathol Commun. 2019 Mar 13;7:39. doi: 10.1186/s40478-019-0694-6 (PMC6416930; doi:10.1186/s40478-019-0694-6)
Supplement: Supplementary file 1 — Table S3. Antibody information. (DOCX 16 kb) [file 40478_2019_694_MOESM1_ESM.docx]

**Table S3: antibodies**

| **Ab name** | **Host** | **Company** | **Cat.nr** | **Dilution** |
| --- | --- | --- | --- | --- |
| Rad23B | mouse | GeneTex | GTX16485 | 1:50 |
| Rad23B | rabbit | Abcam | ab86781 | 1:250 |
| XPC | rabbit | Bethyl | A301-122A | 1:100 |
| 20S | rabbit | ENZO life sciences | BML-PW8155-0025 | 1:100 |
| NGly | rabbit | Novus | NBP1-83793 | 1:100 |
| Ataxin-3 | mouse | Millipore | MAP5360 | 1:1000 |
| ADARB2 | rabbit | Atlas Antibodies | HPA031333 | 1:100 |
| Pur-alpha | rabbit | LS Bio | LS-B6784 | 1:200 |
| FMRP | mouse | N/A | N/A | 1:100 |
| Ran-GAP | rabbit | Abcam | Ab4784 | 1:1000 |
| Poly-GR | mouse | LifeTein Services | N/A | 1:4000 |
| Poly-PR | mouse | LifeTein Services | N/A | 1:500 |
| Poly-GP | rabbit | Bio Connect Life Sciences | 24494-1-AP | 1:250 |
| Poly-GA | mouse | Millipore, clone 5E9 | MABN889 | 1:500 |
| Poly-PA | mouse | Gift from Petrucelli | n.a. | 1:2500 |
| pTDP-43 | mouse | Cosmo bio | CAC-TIP-PTD-M01 | 1:1000 |
| p62 | mouse | BD Biosciences | 610833 | 1:100 |
| XPA | rabbit | Santa Cruz | sc-853 | 1:50 |
| XPB | rabbit | Santa Cruz | sc-293 | 1:1000 |
| XPC | rabbit | Bethyl | A301-121A | 1:1000 |
| XPF | mouse | Santa Cruz | sc-136153 | 1:1000 |
| XPG | rabbit | Bethyl | A301-484A | 1:100 |
| CPD | mouse | CosmoBio | TDM-2 | 1:1000 |
| poly-HRP anti Ms/Rb IgG | goat | Immunologic | DPV055HRP | undiluted |
| anti-mouse HRP | goat | DAKO | P0260 | 1:100 |
| anti-rabbit HRP | goat | DAKO | P0217 | 1:100 |
| anti-mouse Cy2 | goat | Jackson | 715-255-150 | 1:100 |
| anti-rabbit Cy3 | goat | Jackson | 711-165-152 | 1:100 |
