# Supplementary material for: HR23B pathology preferentially co-localizes with p62, pTDP-43 and poly-GA in C9ORF72-linked frontotemporal dementia and amyotrophic lateral sclerosis
Source: Acta Neuropathol Commun. 2019 Mar 13;7:39. doi: 10.1186/s40478-019-0694-6 (PMC6416930; doi:10.1186/s40478-019-0694-6)
Supplement: Supplementary file 2 — Table S1. Neuropathological scores of C9ORF72 FTD patients. Neuronal loss score was based on hematoxylin and eosin (HE) staining and pathological report and scored as absent (0), mild (1), moderate (2) or severe (3). Pathological scores were based on the degree of pathology as absent (0), rare (1), occasional (2), moderate (3), or numerous (4). Brain areas: F = frontal cortex, T = temporal cortex, M = motor cortex, H = hippocampus dentate gyrus, C = cerebellum. NCI = neuronal cytoplasmic inclusion, NII = neuronal intranuclear inclusion, DNs = dystrophic neurites. (DOCX 23 kb) [file 40478_2019_694_MOESM2_ESM.docx]

| Patient ID | | 1 | | | | | 2 | | | | | 3 | | | | | 4 | | | | | 5 | | | | |
| --- | --- | --- | --- | --- | --- | --- | --- | --- | --- | --- | --- | --- | --- | --- | --- | --- | --- | --- | --- | --- | --- | --- | --- | --- | --- | --- |
| Diagnosis | | ***C9ORF72* FTD** | | | | | ***C9ORF72* FTD** | | | | | ***C9ORF72* FTD** | | | | | ***C9ORF72* FTD** | | | | | ***C9ORF72* FTD** | | | | |
| Brain area | | **F** | **T** | **M** | **H** | **C** | **F** | **T** | **M** | **H** | **C** | **F** | **T** | **M** | **H** | **C** | **F** | **T** | **M** | **H** | **C** | **F** | **T** | **M** | **H** | **C** |
| Neuronal loss score | | **2** | **2** | **1** | **2** | **0** | **1** | **3** | **0** | **2** | **0** | **2** | **2** | **1** | **3** | **0** | **1** | **2** | **0** | **2** | **0** | **2** | **2** | **1** | **3** | **1** |
| P62 score | **Total** | **4** | **4** | **4** | **4** | **4** | **4** | **4** | **2** | **4** | **3** | **4** | **3** | **3** | **4** | **3** | **3** | **3** | **3** | **3** | **4** | **4** | **4** | **2** | **3** | **4** |
|  | NCI | 4 | 4 | 4 | 4 | 4 | 4 | 3 | 2 | 4 | 3 | 3 | 2 | 2 | 4 | 3 | 3 | 3 | 3 | 3 | 4 | 4 | 4 | 2 | 3 | 4 |
|  | NII | 2 | 1 | 0 | 0 | 1 | 1 | 1 | 1 | 0 | 1 | 0 | 0 | 0 | 0 | 0 | 1 | 1 | 1 | 1 | 1 | 1 | 1 | 1 | 0 | 2 |
|  | DNs | 3 | 3 | 2 | 0 | 0 | 3 | 4 | 1 | 0 | 0 | 4 | 4 | 3 | 0 | 0 | 3 | 3 | 2 | 0 | 3 | 4 | 3 | 1 | 0 | 0 |
| pTDP-43 score | **Total** | **3** | **3** | **3** | **3** | **0** | **3** | **3** | **1** | **4** | **0** | **2** | **3** | **3** | **4** | **0** | **3** | **3** | **3** | **2** | **0** | **3** | **4** | **1** | **3** | **0** |
|  | NCI | 3 | 3 | 3 | 3 | 0 | 3 | 2 | 1 | 4 | 0 | 1 | 3 | 3 | 4 | 0 | 3 | 3 | 3 | 2 | 0 | 3 | 4 | 1 | 3 | 0 |
|  | NII | 1 | 1 | 1 | 0 | 0 | 1 | 1 | 1 | 0 | 0 | 0 | 0 | 0 | 0 | 0 | 1 | 1 | 1 | 0 | 0 | 1 | 1 | 1 | 0 | 0 |
|  | DNs | 4 | 3 | 2 | 0 | 0 | 3 | 3 | 0 | 0 | 0 | 3 | 1 | 1 | 0 | 0 | 3 | 3 | 2 | 0 | 0 | 3 | 3 | 1 | 0 | 0 |
| HR23B score | **Total** | **1** | **2** | **1** | **1** | **1** | **1** | **2** | **1** | **2** | **1** | **1** | **2** | **1** | **1** | **1** | **1** | **2** | **1** | **1** | **1** | **1** | **2** | **1** | **1** | **2** |
|  | NCI | 1 | 1 | 1 | 1 | 1 | 1 | 1 | 1 | 2 | 1 | 1 | 1 | 0 | 1 | 1 | 1 | 1 | 1 | 1 | 1 | 1 | 1 | 1 | 1 | 2 |
|  | NII | 0 | 1 | 1 | 0 | 1 | 0 | 0 | 0 | 0 | 1 | 1 | 1 | 0 | 0 | 0 | 1 | 1 | 1 | 1 | 1 | 1 | 0 | 0 | 0 | 1 |
|  | DNs | 1 | 3 | 1 | 0 | 0 | 1 | 2 | 1 | 0 | 0 | 2 | 3 | 1 | 0 | 0 | 1 | 3 | 1 | 0 | 0 | 2 | 2 | 1 | 0 | 0 |
