# Supplementary figures and images for: HR23B pathology preferentially co-localizes with p62, pTDP-43 and poly-GA in C9ORF72-linked frontotemporal dementia and amyotrophic lateral sclerosis
Source: Acta Neuropathol Commun. 2019 Mar 13;7:39. doi: 10.1186/s40478-019-0694-6 (PMC6416930; doi:10.1186/s40478-019-0694-6)

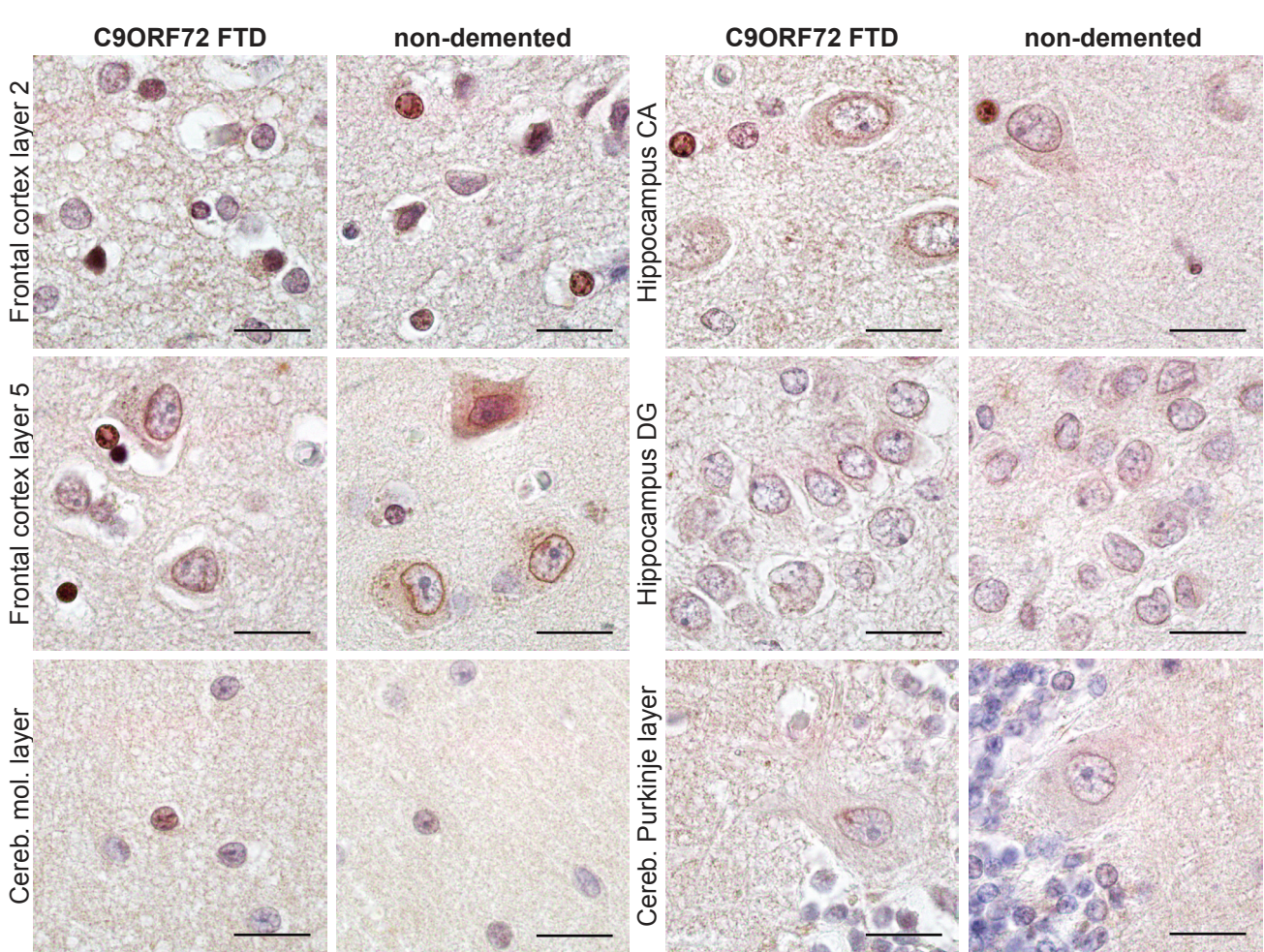

Supplement: Supplementary file 3 — Figure S1. Ran-GAP staining in C9FTD cases and non-demented controls. Ran-GAP is predominantly localized to the nucleus and nuclear membrane. Unevenly shaped nuclear membranes occur in both C9ORF72 FTD cases (n = 5) and non-demented controls (n = 3). All scale bars are 20 μm. (PDF 2202 kb) [file 40478_2019_694_MOESM3_ESM.pdf]

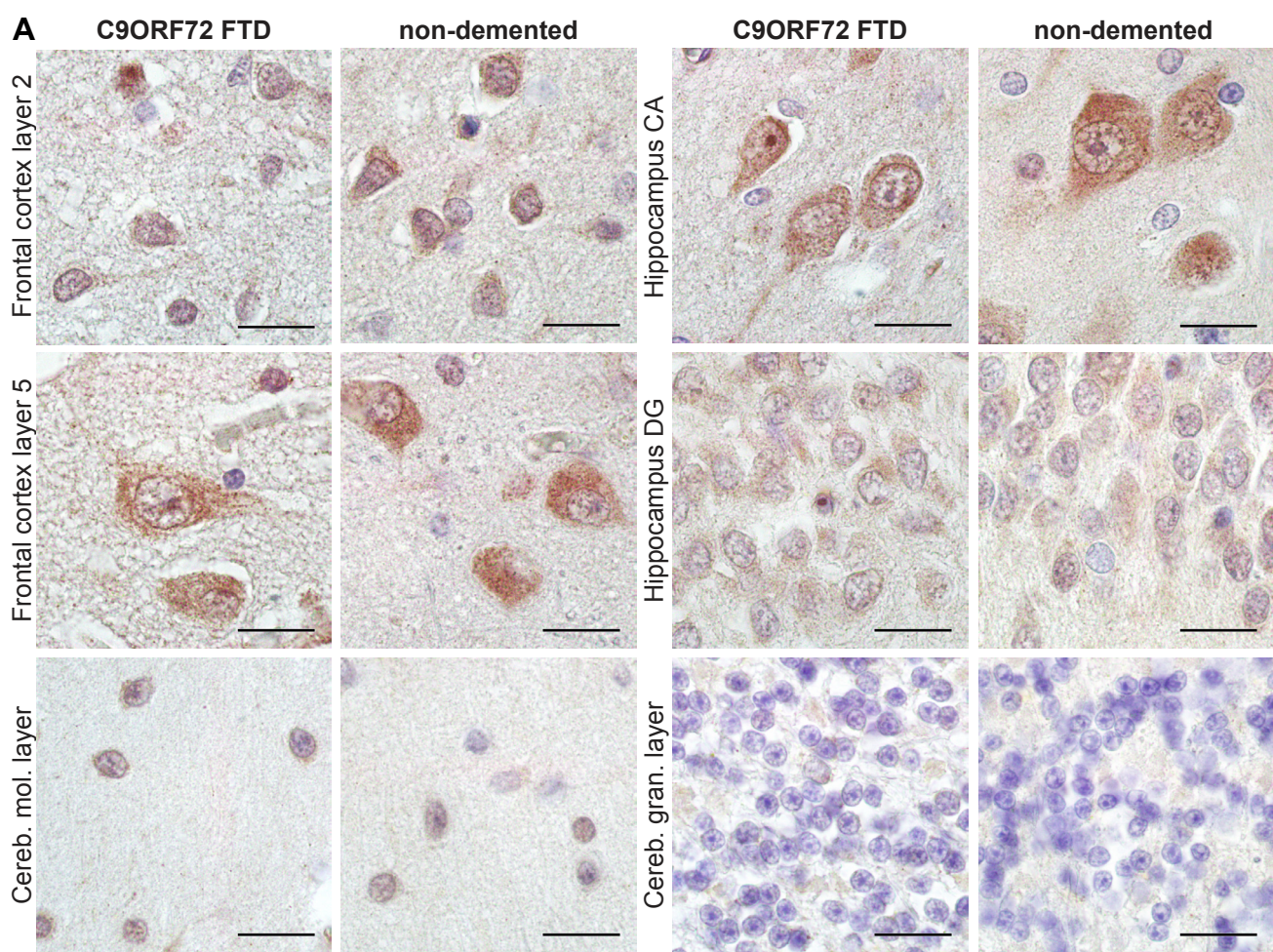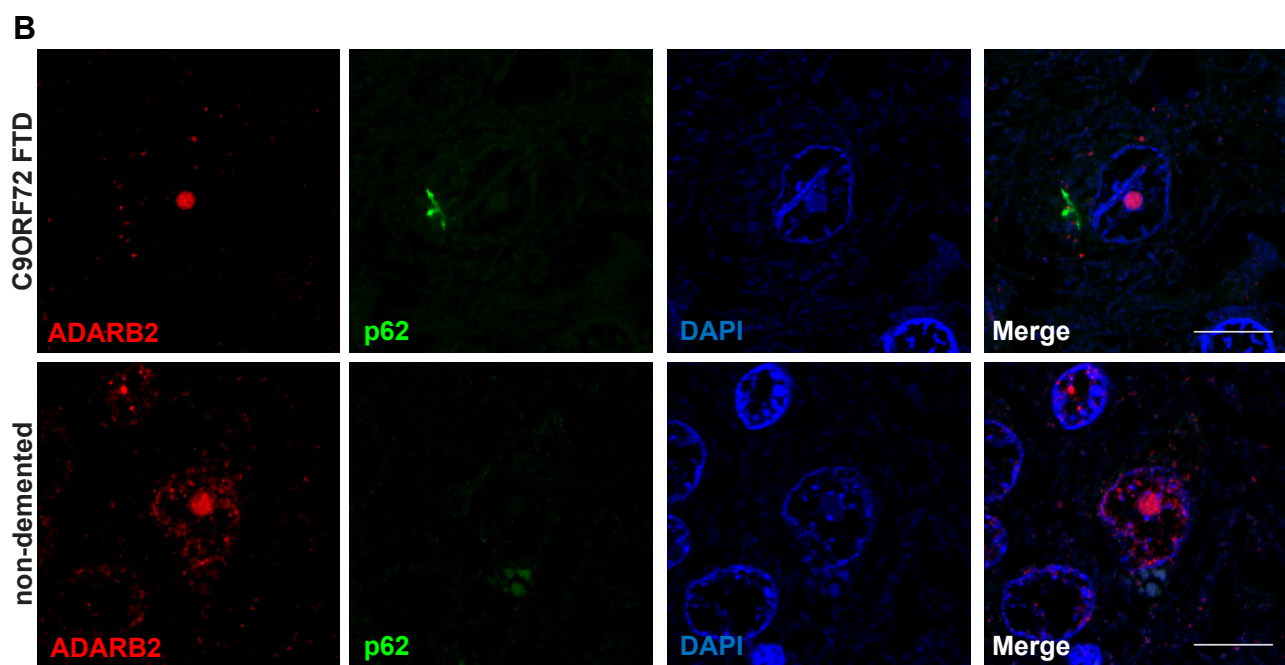

Supplement: Supplementary file 4 — Figure S2. ADARB2 staining in C9FTD cases and non-demented controls. A) Staining of ADARB2 in C9ORF72 FTD cases (n = 5) and non-demented control (n = 3) post-mortem brain sections shows some intranuclear inclusions in hippocampus CA and DG. All scale bars are 20 μm B) Immunofluorescence staining of ADARB2 (red) and p62 (green) in hippocampal dentate gyrus reveals ADARB2 punctuated staining and some intranuclear inclusions in both C9ORF72 FTD cases (n = 5) and non-demented controls (n = 3). Scale bars in fluorescent pictures are 10 μm. (PDF 2721 kb) [file 40478_2019_694_MOESM4_ESM.pdf]

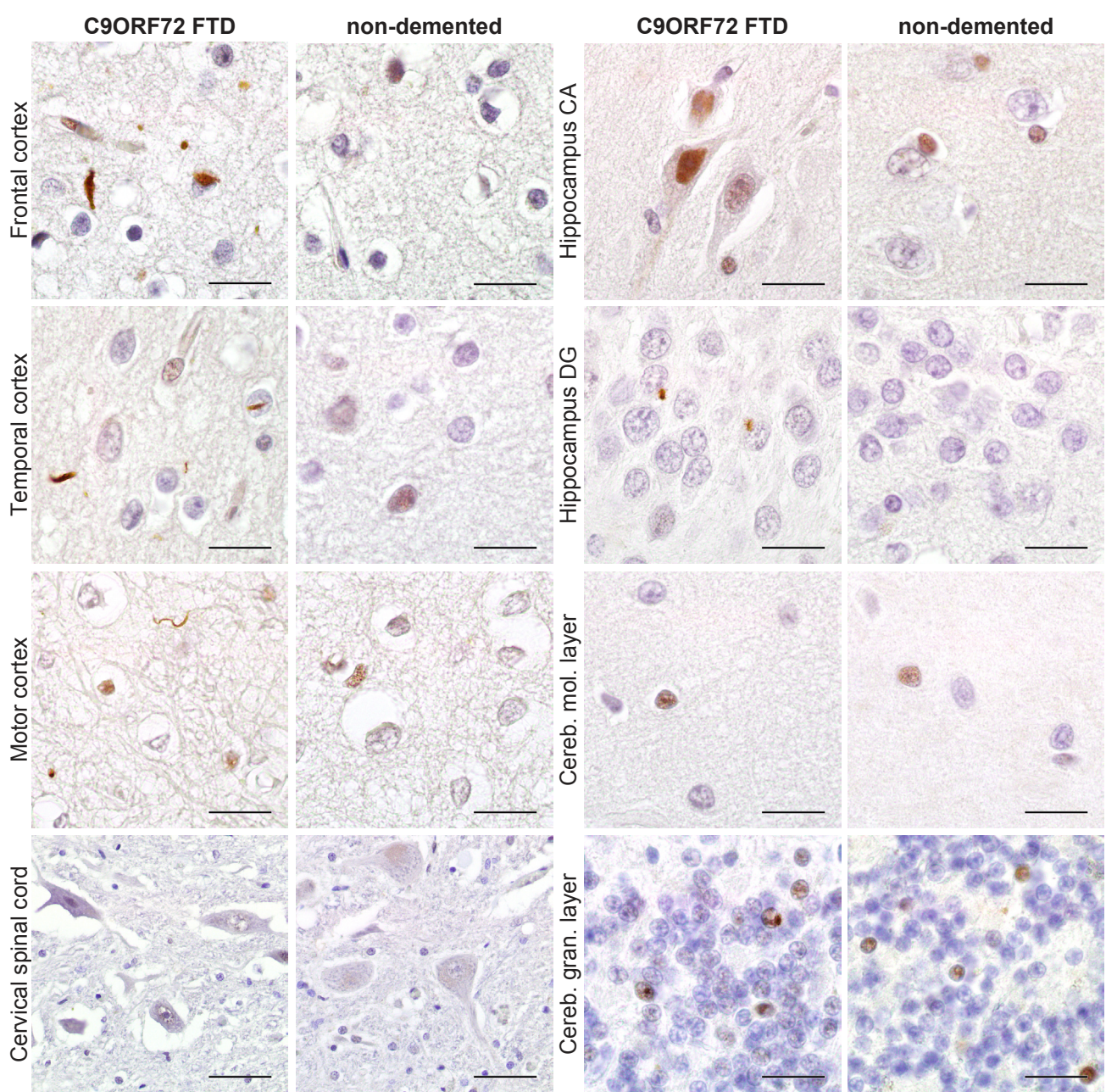

Supplement: Supplementary file 5 — Figure S5. HR23B pathology in different brain areas of C9FTD cases. Staining of HR23B in several brain areas of C9FTD cases and non-demented controls. Pathology burden was highest in cortices (frontal, temporal and motor) and was mostly cytoplasmic (inclusions and neuropils) and intranuclear (cateye). Hippocampus dentate gyrus (DG) harbors perinuclear inclusions, and hippocampus cornu ammonis (CA) had some cells with strong nuclear staining. Pathology was low in cerebellum granular layer with only some nuclear and perinuclear inclusions and almost absent in cerebellum molecular layer. Our C9FTD cases did not show HR23B pathology in spinal cord neurons. All scale bars are 20 μm (PDF 2260 kb) [file 40478_2019_694_MOESM5_ESM.pdf]

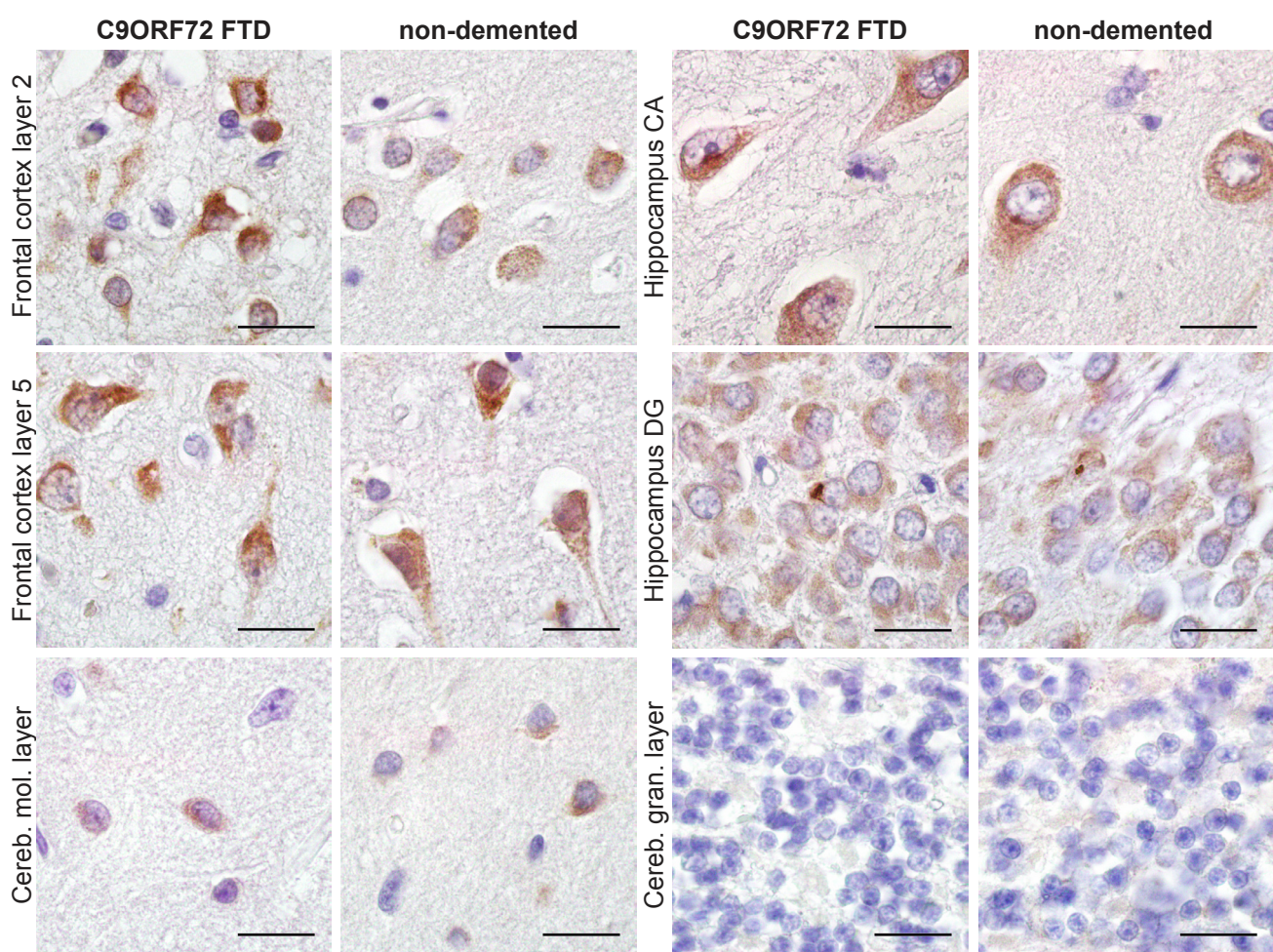

Supplement: Supplementary file 6 — Figure S3. FMRP staining in C9FTD cases and non-demented controls. FMRP staining does not reveal any differences between C9ORF72 FTD cases (n = 5) and non-demented control (n = 3) post-mortem brain sections. Occasional inclusions are found in the hippocampus dentate gyrus in both C9FTD cases and controls. All scale bars are 20 μm. (PDF 1883 kb) [file 40478_2019_694_MOESM6_ESM.pdf]

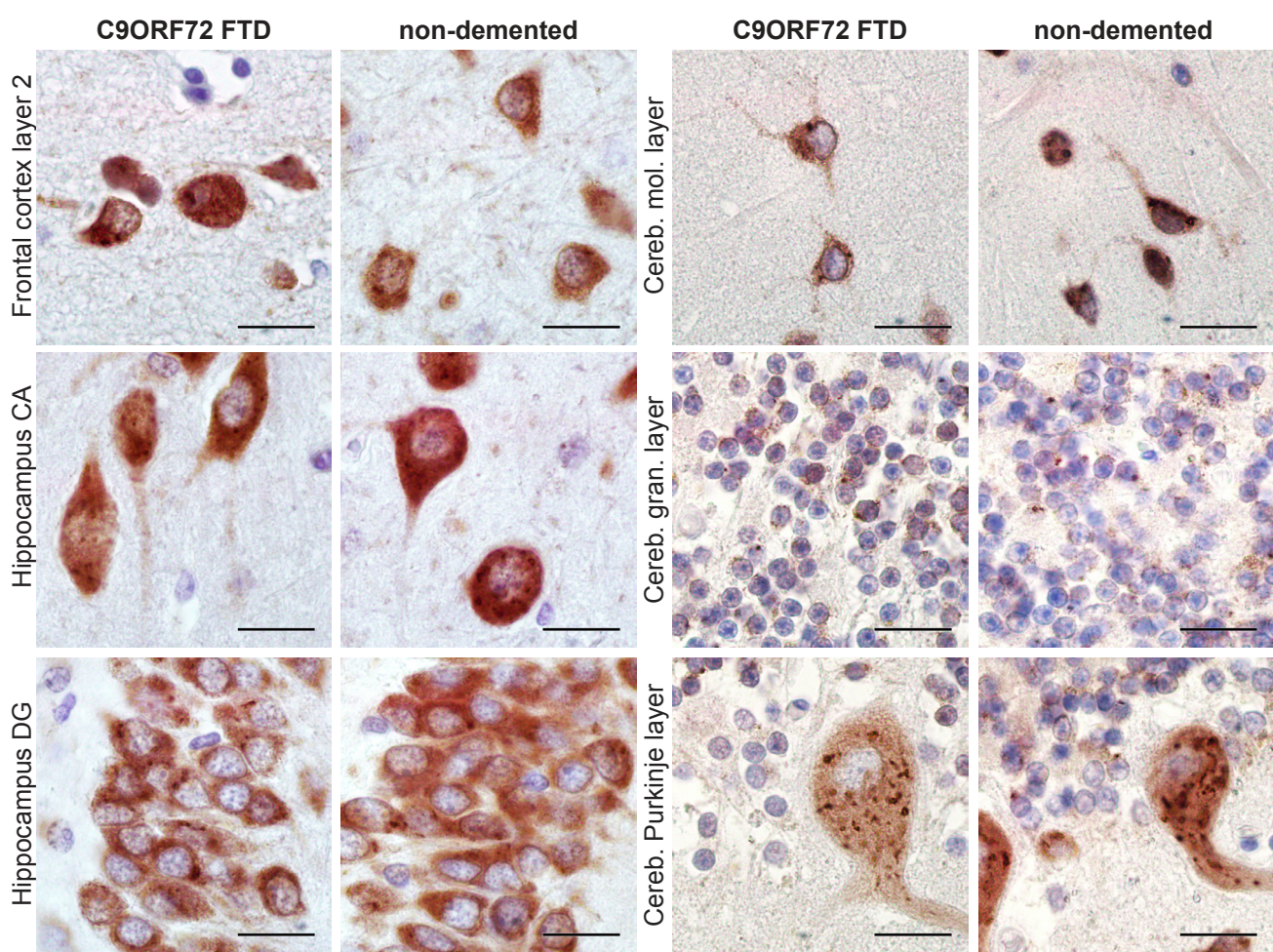

Supplement: Supplementary file 7 — Figure S4 Pur-alpha staining in C9FTD cases and non-demented controls. Pur-alpha staining reveals abundant stress granules in both C9ORF72 FTD cases (n = 5) and non-demented controls (n = 3) post-mortem brain sections. All scale bars are 20 μm. (PDF 1826 kb) [file 40478_2019_694_MOESM7_ESM.pdf]

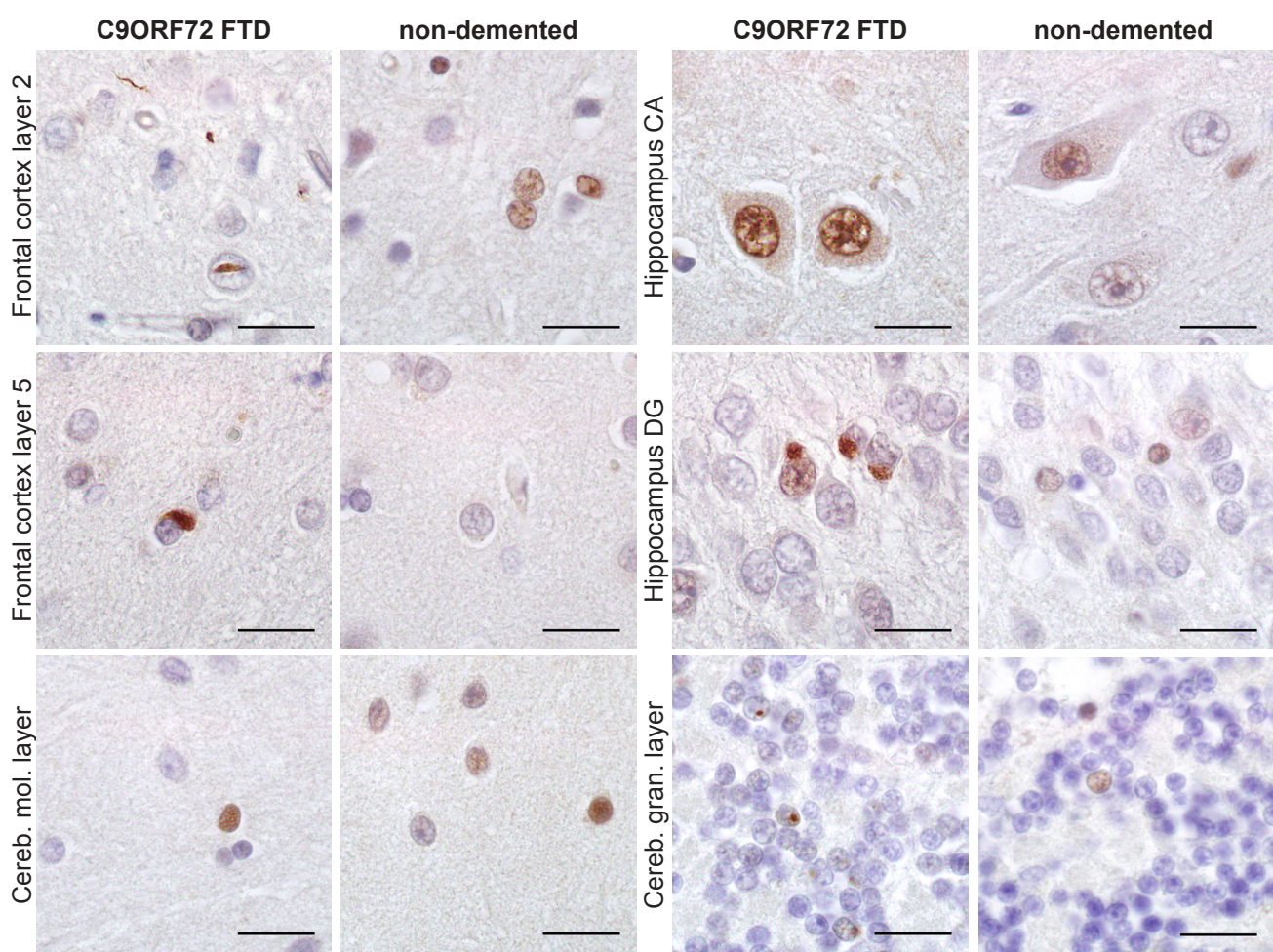

Supplement: Supplementary file 8 — Figure S6 Validation of HR23B pathology by a second independent antibody. HR23B staining using Abcam antibody in C9ORF72 FTD cases (n = 5) and non-demented control (n = 3) post-mortem brain sections. Staining pattern is consistent with HR23B GeneTex antibody (see Figs. 1 and 2). All scale bars are 20 μm (PDF 1543 kb) [file 40478_2019_694_MOESM8_ESM.pdf]

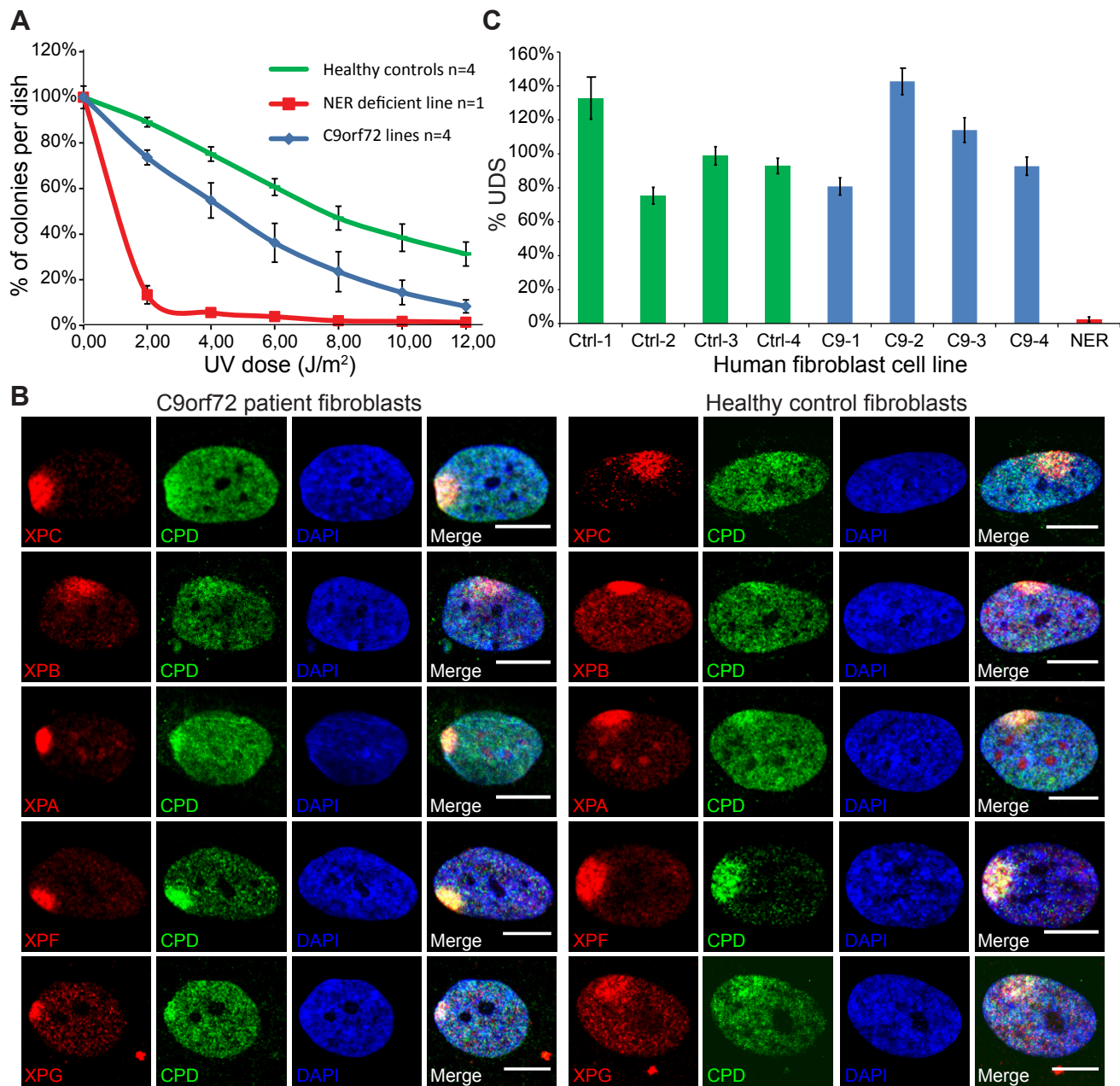

Supplement: Supplementary file 10 — Figure S7. Nucleotide excision repair is not affected in C9ORF72 human fibroblasts. A) Dose-response curve for 4 healthy control human fibroblast lines (81E253, 86E1375, 06E0717 and 99E0774) and 4 C9ORF72 human fibroblast lines (13E634, 13E659, 17E0225, 17E0278) and the NER deficient XP25RO human fibroblast line treated with increasing dose of UV-C light (0–12 J/m2). B) Immunofluorescence staining showing the recruitment of NER factors XPC, XPB, XPA, XPF and XPG to local DNA damage (visualized by CPD antibody), induced by 60 J/m2 UV-C irradiation through a microporous filter. Fibroblast lines used for pictures: 13E634, 13E659 and 81E253 C) Human fibroblasts lines were treated with 16 J/m2 UV-C light and incubated with EdU for 1 h to measure unscheduled DNA synthesis (UDS) as measure of DNA repair. The NER-deficient XPC25RO cell line is shown as negative control. Ctrl 1–4 are lines 81E253, 86E1375, 06E0717 and 99E0774 in this order. C9 1–4 are lines 13E634, 13E659, 17E0225, 17E0278 in this order. (PDF 934 kb) [file 40478_2019_694_MOESM10_ESM.pdf]
