# Supplementary material for: HR23B pathology preferentially co-localizes with p62, pTDP-43 and poly-GA in C9ORF72-linked frontotemporal dementia and amyotrophic lateral sclerosis
Source: Acta Neuropathol Commun. 2019 Mar 13;7:39. doi: 10.1186/s40478-019-0694-6 (PMC6416930; doi:10.1186/s40478-019-0694-6)
Supplement: Supplementary file 9 — Table S2. Co-localization of HR23B with DPRs/pTDP-43/p62 differs between brain areas. The percentage is the amount of HR23B inclusions also positive for other pathological hallmarks (not the other way around). For example 11/153 for poly-GA in C9FTD patient 1 means that out of 153 HR23B inclusions, 11 were also positive forpoly- GA, which is 7%. F = frontal cortex. H = hippocampus dentate gyrus. *1 = All co-localizations are fibrils of poly-GP and HR23B in frontal cortex. Perinuclear inclusions of poly-GP did not stain positive for HR23B. *2 = In total 2 poly-PA inclusions have been found in frontal cortex of 5 C9FTD patients, too less to quantify. We therefore state N/A = non-applicable in this table. *3 = Non-demented cases had some p62 and some HR23B inclusions per person per section, which sometimes overlapped. No pTDP-43 inclusions were found so no co-localization of pTDP-43 with HR23B in non-demented cases. (DOCX 19 kb) [file 40478_2019_694_MOESM9_ESM.docx]

|  |  |  | **HR23B +** | | | | | | |
| --- | --- | --- | --- | --- | --- | --- | --- | --- | --- |
| **Gene & diagnosis** | **ID number** | **Brain area** | **Poly- GA** | **Poly-GP*^1^** | **Poly-GR** | **Poly-PR** | **Poly-PA *^2^** | **pTDP-43** | **p62** |
|  |  |  |  |  |  |  |  |  |  |
| *C9ORF72*  FTD | 1 | F | 11/153 = 7% | 0/103 = 0% | 1/109 = 1% | 0/150 = 0% | N/A | 19/121 = 16 % | 72/105 = 68% |
|  |  | H | 77/90  = 85% | 3/91 = 3,2% | 20/191 = 10% | 2/285 = 0.7% | N/A | 32/104 = 31% | 118/150 = 78% |
|  | 2 | F | 7/62 = 11% | 3/102 = 3% | 3/102 = 3% | 1/80 = 1,25% | N/A | 42/101 = 42 % | 72/107 = 67% |
|  |  | H | 62/135 = 46% | 3/173 = 1,7% | 9/212 = 4,3% | 3/469 = 0,6% | N/A | 39/111 = 35% | 140/156 = 90% |
|  | 3 | F | 11/180 = 6% | 0/107 = 0% | 1/237 = 0,4% | 0/120 = 0% | N/A | 35/140 = 25% | 79/106 = 75% |
|  |  | H | 52/97 = 54% | 5/115 = 4,3% | 13/99 = 13% | 1/176 = 0,5% | N/A | 31/106 = 30% | 65/74 =  88% |
|  | 4 | F | 6/103 = 6% | 1/105 = 1% | 0/115 = 0% | 2/100 = 2% | N/A | 26/117 = 22% | 63/116 = 54% |
|  |  | H | 42/72 = 58% | 5/102 = 4,9% | 16/105 = 15% | 4/154 = 2,6% | N/A | 39/115 = 34 % | 92/102 = 90% |
|  | 5 | F | 5/175 = 3% | 0/100 = 0% | 1/131 = 0,8% | 0/160 = 0% | N/A | 9/105 =  9% | 92/136 = 68% |
|  |  | H | 54/90 = 60% | 10/107 = 9,3% | 10/105 = 9,5% | 0/68 = 0% | N/A | 22/100 = 22% | 61/66 =  92% |
|  |  |  |  |  |  |  |  |  |  |
| *GRN*  FTD | 6 | F | 0% | 0% | 0% | 0% | N/A | 59/178 = 33% | 118/157 = 75% |
|  |  | H | 0% | 0% | 0% | 0% | N/A | 5/33 = 15% | 5/5 =  100% |
|  | 7 | F | 0% | 0% | 0% | 0% | N/A | 61/125 = 49% | 100/154 = 65% |
|  |  | H | 0% | 0% | 0% | 0% | N/A | 1/13=  8% | 3/3 =  100% |
|  |  |  |  |  |  |  |  |  |  |
| Non-demented cases *^3^ | 16 | F | 0% | 0% | 0% | 0% | N/A | 0% | ½ = 50% |
|  |  | H | 0% | 0% | 0% | 0% | N/A | 0% | 0% |
|  | 17 | F | 0% | 0% | 0% | 0% | N/A | 0% | 1/3 = 33% |
|  |  | H | 0% | 0% | 0% | 0% | N/A | 0% | 1/1=100% |
|  | 18 | F | 0% | 0% | 0% | 0% | N/A | 0% | 2/2 = 100% |
|  |  | H | 0% | 0% | 0% | 0% | N/A | 0% | 1/1=100% |
|  |  |  |  |  |  |  |  |  |  |
